# Dunglison’s Practice of Medicine

**Published:** 1844-07

**Authors:** 


DUNGLISON'S PRACTICE OF MEDICINE.

In the short space of two years, a second edition of Dr. Dunglison's Treatise on Special Pathology and Therapeutics has been called for, and is now before the public in the neat and tasteful dress in which Lea & Blanchard issue all their valuable publications. We do not notice the fact for the purpose of passing any studied eulogy upon this work, which is now too well known to the profession to need the commendation of the press. The author has cause to be gratified with its success, and, we have no doubt, expresses himself with sincerity when he says, that it will incite him "to untiring exertion to render every succeeding edition more and more valuable." A cursory examination will satisfy any one, that great labor has been bestowed upon these volumes, and on a careful perusal it will be seen that they exhibit the present state of our knowledge relative to special pathology and therapeutics. The work is justly a great favorite with students of medicine, whose exigencies the learned author seems especially to have consulted in its preparation. Y.

